# Sputum culture contamination and associated characteristics in a diagnostic clinical trial, Papua New Guinea

**DOI:** 10.5588/pha.24.0029

**Published:** 2024-12-01

**Authors:** ND. Tenakanai, J.K. Banamu, Y. Lin, D. Walsh, R. Simbil, M. Globan, A. Penn-Nicholson, P. du Cros, J. Greig

**Affiliations:** ^1^ Burnet Institute, Melbourne, Australia;; ^2^ Central Public Health Laboratory, PNG National Department of Health (NDoH), Port Moresby, PNG;; ^3^ Papua New Guinea Institute of Medical Research (PNGIMR), Goroka, PNG;; ^4^ Mycobacterium Reference Laboratory, Victorian Infectious Diseases Reference Laboratory at the Peter Doherty Institute, Melbourne, Australia;; ^5^ FIND, Geneva, Switzerland.

**Keywords:** self-collected sputum, specimen transport, tuberculosis

## Abstract

**SETTING:**

Papua New Guinea is a high-burden multidrug-resistant TB (MDR/RR-TB) country that reports low rates of bacteriological confirmation. Sputum drug susceptibility testing for second-line drugs is important. Access to culture is limited.

**OBJECTIVE:**

To evaluate the prevalence of mycobacterial sputum sample culture contamination and determine factors associated with contamination.

**DESIGN:**

A retrospective analysis of data from a TB diagnostic accuracy study that used culture as the diagnostic reference standard. Data included characteristics of people with presumptive pulmonary TB who provided sputum, sputum quality and culture results.

**RESULTS:**

Sputum (1–3 samples) was collected from 174 adults. The median age was 33 years (IQR 24–47). Of 337 samples sent for culture, 28% were contaminated. Contamination was strongly associated with samples self-collected by participants outside clinic hours (aOR 5.69; 95% CI 2.62–12.38). Contamination risk increased with delays in shipping to the reference laboratory (aOR 1.19 per day, 95% CI 1.10–1.29) beyond the minimum 3 days. Contamination was less frequent among people aged 35–44 years compared to 18–24 years (aOR 0.27, 95% CI 0.10–0.73). Sputum quality was not associated with culture contamination.

**CONCLUSION:**

Culture contamination could be reduced using spot sputum collection, expedited submission to laboratories and faster shipping when required.

## Abstract (fr)

**CONTEXTE:**

La Papouasie-Nouvelle-Guinée est un pays où la TB multirésistante (MDR-TB) est largement répandue, et les taux de confirmation bactériologique y sont faibles. Il est essentiel de réaliser des tests de sensibilité des expectorations aux médicaments de deuxième ligne. L'accès à la culture est restreint.

**OBJECTIF:**

Évaluer la fréquence de la contamination des cultures d'échantillons d'expectorations mycobactériennes et identifier les facteurs liés à cette contamination.

**MÉTHODES:**

Analyse rétrospective des données d'une recherche concernant la précision du diagnostic de la TB, ayant utilisé la culture comme référence pour établir le diagnostic. Les données incluaient les caractéristiques des individus présentant une TB pulmonaire présumée ayant fourni des échantillons d'expectorations, ainsi que la qualité de ces échantillons et les résultats des cultures.

**RÉSULTATS:**

Des échantillons d'expectorations (1 à 3 échantillons) ont été recueillies auprès de 174 adultes. L'âge médian des participants était de 33 ans (IQR 24–47). Parmi les 337 échantillons envoyés pour culture, 28% se sont révélés contaminés. Cette contamination était fortement liée aux échantillons prélevés par les participants eux-mêmes en dehors des heures d'ouverture des cliniques, avec un odds ratio ajusté (ORa) de 5,69 et un intervalle de confiance à 95% (IC 95%) de 2,62 à 12,38. De plus, le risque de contamination augmentait avec le temps d'expédition vers le laboratoire de référence, avec un ORa de 1,19 par jour (IC 95% 1,10–1,29) au-delà d'un délai minimum de 3 jours. Il a également été observé que la contamination était moins fréquente chez les individus âgés de 35 à 44 ans par rapport à ceux de 18 à 24 ans, avec un ORa de 0,27 (IC 95% 0,10–0,73). Enfin, la qualité des expectorations ne semblait pas avoir d'association avec la contamination par culture.

**CONCLUSION:**

La contamination par culture pourrait être réduite par la collecte régulière d'expectorations, une transmission rapide aux laboratoires et un envoi plus efficace en cas de nécessité.

TB is a major cause of morbidity and mortality globally and in Papua New Guinea (PNG).^1^ In PNG in 2022, there were 36,644 TB case notifications; only 38% of pulmonary TB was bacteriologically confirmed.^2^ Bacteriological confirmation of TB, whenever possible, is important for optimising treatment outcomes, particularly for drug-resistant TB. The expansion of the GeneXpert system (Cepheid, Sunnyvale, CA, USA) for TB testing in PNG began in 2012 and 2013. By the end of 2020, this technology was operational in all 22 provinces, with 70 testing sites established nationwide. This has improved overall case detection with bacteriological confirmation, increased rifampicin-resistant TB (RR-TB) diagnoses, and allowed for earlier effective treatment. However, mycobacterial culture remains the ‘gold standard’ diagnostic approach.

In routine programmatic practice, shipped samples are predominantly from people with microbiologically confirmed RR-TB detected by Xpert^®^ MTB/RIF (or Ultra) and some from people with clinically presumptive RR-TB whose samples are Xpert MTB-negative.^3,4^ These cases require the culture to allow phenotypic drug susceptibility testing (pDST) to detect resistance profiles to a broader range of drugs than currently detected by rapid molecular diagnostics. Samples have also been sent for culture for research studies as the reference standard for evaluating a diagnostic test, as was done during the evaluation of the Truenat MTB Assay (Molbio Diagnostics, Bangalore, India).^5,6^

There are challenges for efficient and timely transport of sputum samples in PNG, leading to long delays in receiving results that inform treatment decisions.^7,8^ Infrastructure and capacity challenges at the only culture facility render culture unavailable in PNG. Samples have to be shipped to mycobacterial reference laboratories in Australia. Handling and storage conditions before and during shipping are known to impact the quality and yield of the specimen.^8^ Sputum culture contamination constitutes the overgrowth of normal flora. Sample contamination may delay or prevent bacteriological confirmation and performance of pDST.

PNG was one site in a multi-national study that evaluated Truenat MTB, MTB Plus and RIF-Dx assays (Molbio) for TB diagnosis compared to solid and liquid culture.^5^ The percentage of samples from PNG with culture contamination using solid and liquid media was higher than acceptable proportions of respectively <5% and <10%.^7,8^ As contaminated culture results were excluded from the trial analysis, the rates of sputum contamination for PNG were not reported. We therefore aimed to review contamination data and evaluate factors associated with contaminated culture results.

## METHODS

### Study setting, design and population

Port Moresby General Hospital (PMGH) is the largest hospital in PNG. It provides tertiary specialist services and outpatient and inpatient diagnostic and treatment services for the surrounding population of Port Moresby, the capital of PNG.

We conducted a retrospective descriptive cohort study using data from sputum culture results collected during a TB diagnostic clinical trial. Full multi-site results and methods have been published.^5^ At the PNG study site (PMGH), samples were collected from adults with presumptive pulmonary TB who presented to the TB clinic from July 2019 to March 2020 and had not received TB treatment interventions in the prior 6 months. Initial sample decontamination and tests for the diagnostic clinical trial were performed in a laboratory located a short walk from the TB clinic.

Three sputum samples were collected per participant as specified in the diagnostic clinical trial protocol and as routinely done in PNG at the time. Trained staff explained sputum collection instructions using an illustrated flip chart and verbal explanation. Instructions included pre-rinsing the mouth with water. Two observed (spot) sputum samples were collected approximately 20 minutes apart during the initial visit during clinic operating hours. These were put in iceboxes with ice bricks until transported to the study laboratory on the same day. Participants self-collected a third sample, usually at home outside clinic operating hours, with instructions for cool storage until brought to the TB outpatient clinic during operating hours. These were stored with spot samples until taken to the study laboratory on the same day (Table 1). When received at the laboratory, all samples were refrigerated at 2–8°C and handled stringently with good laboratory practice throughout all procedures. Smears were prepared from spot sputa and microscopically assessed for acid-fast bacilli. Pairs of spot sputum samples were combined and homogenised, then subjected to TB testing using the Xpert MTB/RIF and Truenat assays, both before and after undergoing a decontamination process. Sputum was decontaminated using sodium hydroxide and *N*-acetyl-L-cysteine (NaOH NALC-2%);^8^ a portion of this decontaminated sample was refrigerated for culture. Refrigerated samples were batched and sent to the Mycobacterium Reference Laboratory at the Victorian Infectious Diseases Reference Laboratory (VIDRL, Melbourne, Australia) for culture and DST. Two samples were cultured per individual: one combined (spot Sputums 1 + 2) and sputum 3. Samples were cultured on both solid (Löwenstein–Jensen) and liquid media (Mycobacteria Growth Indicator Tube [MGIT], BD, Franklin Lakes, NJ, USA). We report initial culture results for all samples and methods before additional decontamination processes at the reference laboratory. Dates for process steps throughout the trial were recorded. We calculated days between dates of sputum collection, decontamination, and storage in the trial site laboratory and shipment from this laboratory until inoculation onto culture media at the reference laboratory.

**TABLE 1.** Sputum characteristics and collection time by sequential sample number.

Sputum characteristics		Sputum number				
		Total (*n* = 514) *n* (%)	1 (*n* = 174) *n* (%)	2 (*n* = 174) *n* (%)	3 (*n* = 166) *n* (%)	*P*-value*
Temperature, °C, median [IQR]		10 [10–12]	10 [10–12]	10 [10–12]	10 [9–12]	0.32
Time of day collected	Morning	145 (28.2)	68 (39.1)	66 (37.9)	11 (6.6)	<0.001
	Afternoon	178 (34.6)	88 (50.6)	88 (50.6)	2 (1.2)	
	Overnight^†^	191 (37.1)	18 (10.3)	20 (11.5)	153 (92.2)	
Macroscopy	Very viscous	74 (14.4)	22 (12.6)	21 (12.1)	31 (18.7)	0.038
	Viscous	369 (71.8)	128 (73.6)	136 (78.2)	105 (63.3)	
	Not viscous	71 (13.8)	24 (13.8)	17 (9.8)	30 (18.1)	
Purity	No particles	416 (80.9)	141 (81.0)	142 (81.6)	133 (80.1)	0.94
	Contains food particles	98 (19.1)	33 (19.0)	32 (18.4)	33 (19.9)	
Bloodstained	Not bloodstained	468 (91.1)	160 (92.0)	159 (91.4)	149 (89.8)	0.97
	Very bloodstained	16 (3.1)	5 (2.9)	5 (2.9)	6 (3.6)	
	Mildly bloodstained	30 (5.8)	9 (5.2)	10 (5.7)	11 (6.6)	

* Categorical values compared using χ^2^ test and continuous values compared using Kruskal-Wallis test.

† Timing after or before clinic operating hours, that is, after 4 pm until before 9 am the following working day.

IQR = interquartile range.

### Data analysis

Detailed data collected during the diagnostic clinical trial were exported from the centrally managed OpenClinica (OpenClinica, LLC, Needham, MA, USA) database and analysed with Stata/BE-17 (StataCorp, College Station, TX, USA). Analyses compared participant characteristics, their sputum samples and culture results. Descriptive analysis included count and proportion for categorical data, and time differences between process steps were summarised in terms of medians and interquartile ranges (IQRs). Differences in categorical variables were tested using the χ2 test and in continuous values using the Kruskal–Wallis test. The primary outcome was culture-contaminated versus non-contaminated. Characteristics of samples associated with contamination were assessed through multivariable logistic regression with nesting by the participant, with backward selection retaining only variables significantly associated with the outcome. Associations are reported as odds ratios (ORs) with 95% confidence intervals (CIs). *P* < 0.05 was considered statistically significant.

### Ethics approval

Approval to conduct the original clinical trial was obtained from the PNG Medical Research Advisory Committee (MRAC), Central Public Health Laboratory (CPHL), PMGH and the National TB Programme (NTP), Port Moresby, PNG.

## RESULTS

The overall proportion of contaminated specimens was 28% (95/337). The proportion was significantly lower for the combined Sputum 1 + 2 (spot) samples at 17% (29/173) compared to Sputum 3 samples at 40% (66/164). Additional decontamination steps resulted in only 20 pairs of liquid and solid cultures that remained contaminated without a usable culture result for the sample. Our study focused on the 95 initially culture-contaminated samples.

The samples for this study were collected from 174 adults aged between 18 and 70 years who each provided 1–3 sputum samples (total of 514 samples). The median age of the participants was 33 years (IQR 24–47), and 40% were female. Sample characteristics for the three sequentially collected samples were similar, except that most Sputum 3 samples were self-collected by participants outside of clinic operating hours, and a significantly lower proportion were classified as ‘viscous’ on macroscopy examination (*P* = 0.038) (Table 1).

The median time from sputum collection until decontamination in the study laboratory was 1 day (IQR 1–3), and the median time from decontamination and shipping was 6 days (IQR 2–10), with neither duration different for samples later found to be contaminated compared to those not contaminated (Table 2, Figure). The median time from shipment sent until samples were received and inoculated on media at the reference laboratory was 6 days (IQR 5–7); it was only slightly longer (7 days) for contaminated samples, but the difference was significant (*P* < 0.001). Categorising shipping time showed that while the odds of contamination increased incrementally, it rose most substantially after more than 10 days (Table 2).

**TABLE 2.** Characteristics of study participants and sputum samples, proportion of samples with culture contamination, and logistic regression model* for contaminated culture.

Participant or sputum characteristic^†^	Total sputum^†^ *n* (%)	Culture contaminated *n* (%)	OR (95%CI)	*P*-value	aOR (95%CI)	*P*-value
Sex						
Female (*n* = 71)	135 (40.1)	31 (32.6)	1.00			
Male (*n* = 103)	202 (59.9)	64 (67.4)	1.56 (0.94–2.59)	0.086		
Age group, years						
18–24 (*n* =49)	95 (28.2)	35 (36.8)	1.00	0.057	1.00	
25–34 (*n* =41)	81 (24.0)	19 (20.0)	0.53 (0.27–1.02)	0.057	0.57 (0.28–1.18)	0.13
35–44 (*n* =30)	57 (16.9)	7 (7.4)	0.24 (0.10–0.59)	0.0018	0.27 (0.10–0.73)	0.010
45–54 (*n* =29)	56 (16.6)	21 (22.1)	1.03 (0.52–2.04)	0.94	1.21 (0.57–2.57)	0.62
55–70 (*n* =25)	48 (14.2)	13 (13.7)	0.64 (0.30–1.36)	0.24	0.76 (0.33–1.75)	0.51
Sputum microscopy						
Very viscous	52 (15.4)	15 (15.8)	1.00			
Viscous	231 (68.5)	63 (66.3)	0.92 (0.47–1.82)	0.82		
Not viscous	54 (16.0)	17 (17.9)	1.14 (0.49–2.65)	0.77		
Purity						
No particles	273 (81.0)	75 (78.9)	1.00			
Contains food particles	64 (19.0)	20 (21.1)	1.20 (0.66–2.20)	0.55		
Bloodstaining						
Not bloodstained	306 (90.8)	85 (89.5)	1.00			
Very bloodstained	11 (3.3)	4 (4.2)	1.49 (0.42–5.34)	0.54		
Mildly bloodstained	20 (5.9)	6 (6.3)	1.12 (0.41–3.04)	0.83		
Sample number						
1 and 2 (2 spot combined)	173 (51.3)	29 (30.5)	1.00			
3 (self-collected)	164 (48.7)	66 (69.5)	3.71-(2.09–6.58)	<0.0001		
Time of day collected						
Morning	78 (23.1)	10 (10.5)	1.00		1.00	
Afternoon	90 (26.7)	15 (15.8)	1.43 (0.58–3.55)	0.44	1.68 (0.68–4.16)	0.27
Overnight^‡^	169 (50.1)	70 (73.7)	5.27 (2.34–11.85)	0.0001	5.69 (2.62–12.38)	<0.0001
Sputum temperature, °C, median [IQR]	10 [9–12]	11 [10–12]	1.01 (0.92–1.10)	0.88		
Time between collection and decontamination, days, median [IQR]	1 [1–3]	1 [1–3]	0.95 (0.83–1.08)	0.41		
Time between decontamination and shipment, days, median [IQR]	6 [2–10]	5 [2–14]	1.04 (1.00–1.07)	0.051		
Time between shipment and inoculation, days, median [IQR]	6 [5–7]	7 [5–10]	1.18 (1.10–1.27)	<0.0001	1.19 (1.10–1.29)	<0.0001
Time of shipment, days						
3–4	53 (15.9)	8 (8.5)	1.00			
5–7	217 (65.0)	56 (59.6)	1.96 (0.87–4.40)	0.1049		
8–10	21 (6.3)	7 (7.4)	2.81 (0.87–9.14)	0.0855		
>10	43 (12.9)	23 (24.5)	6.47 (2.47–16.92)	0.0001		

* Logistic regression for outcome of contaminated culture, nested by participant.

† Totals for sex and age categories are per included sputum culture, where participants contributed up to two specimens that were cultured because Sputums 1 and 2 were combined and homogenised and a portion sent for culture. Total number of participants contributing these specimens are reported in the first column.

‡ Overnight designates timing after or before clinic operating hours, that is, after 4 pm until before 9 am the following day.

OR = odds ratio; CI = confidence interval; aOR = adjusted OR, IQR = interquartile range.

**FIGURE.**
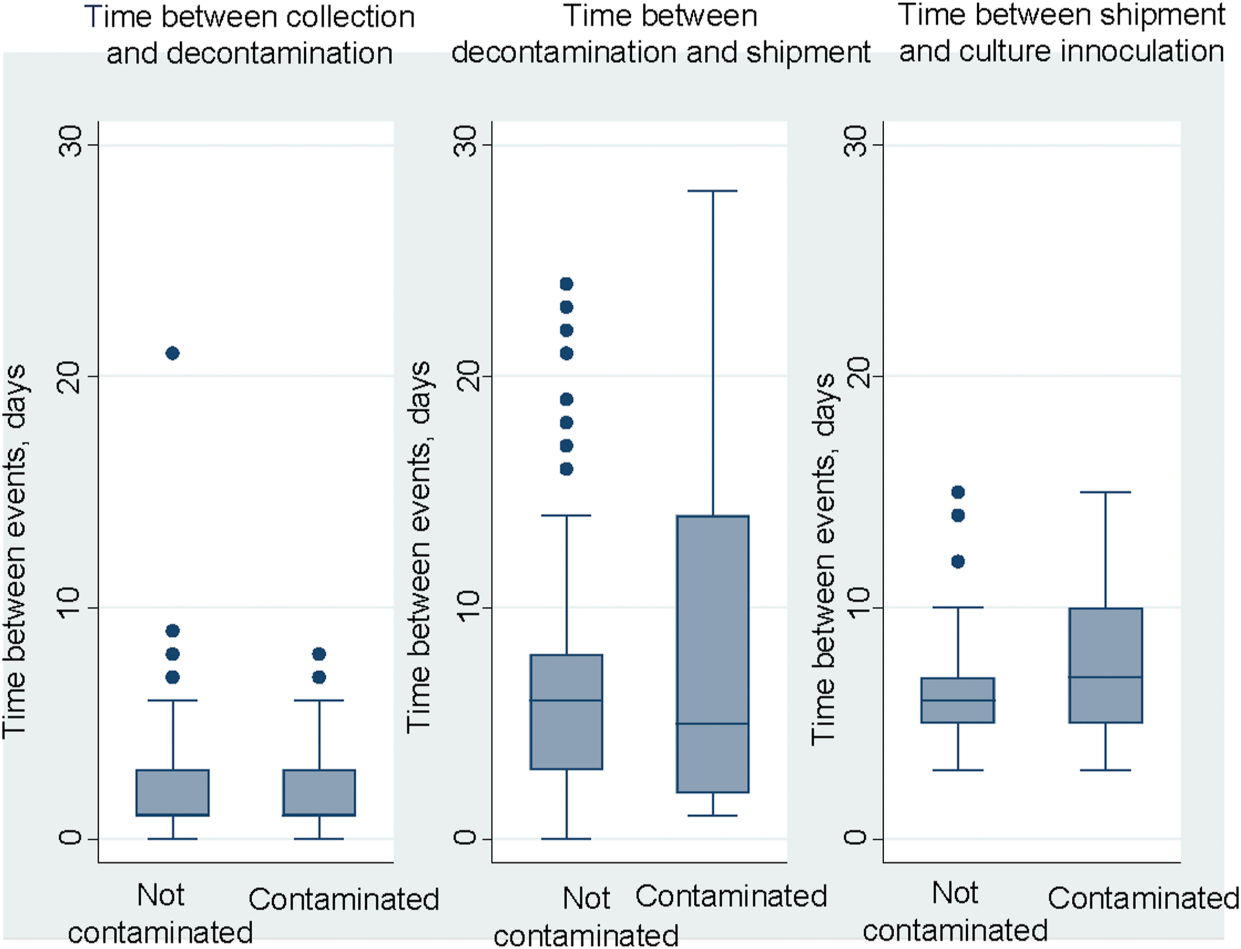
Time in days between sputum collection, decontamination and refrigerated storage in the study laboratory and shipping and inoculation in the reference laboratory, categorised by contamination status of the sample culture.

The factors associated with contaminated cultures revealed no significant associations on univariable analysis for sputum characteristics, except a much higher proportion contaminated with Sputum 3 (Table 2). After adjusting for other variables through nested multivariable logistic regression, collection outside of clinic hours was the most significant factor associated with contaminated culture (adjusted odds ratio [aOR] 5.69, 95% confidence interval [CI] 2.62–12.38) (labelled ‘overnight’ in Table 2). The time delay between shipping the stored samples from the trial site to the reference laboratory and inoculation into the culture was also significantly associated with a contaminated culture result (aOR 1.19, 95% CI 1.10–1.29) for every additional day beyond the shortest duration of 3 days (Table 2).

A slightly higher proportion of contaminated samples were submitted by male participants, but after adjusting for other factors, sex was not significantly associated and was excluded from the model. Multivariable nested regression indicated that a substantially lower proportion of samples were contaminated from participants aged 35–44 years compared to those aged 18–24 years (aOR 0.27, 95% CI 0.10–0.73), but there was no significant difference for other age groups (Table 2).

Samples that were later found to be contaminated on culture were not significantly different in the likelihood of detecting MTB using sputum smear (*P* = 0.09 for Sputum 1; *P* = 0.10 Sputum 2) or Xpert MTB assay (*P* = 0.10 raw combined sputum; *P* = 0.16 decontaminated sputum) at the on-site laboratory.

## DISCUSSION

This study provides original data from PNG that culture contamination is common in sputum samples from people with presumptive TB and has identified potentially modifiable factors associated with contamination. We found that self-collection of sputum samples outside of clinic operating hours was strongly associated with a contaminated culture and incremental increases in odds of contamination with each additional day for shipping from the study laboratory to the reference laboratory.

The proportion of culture contamination in this trial site was higher than that of other trial sites and much higher than acceptable contamination rates for both solid and liquid cultures.^5,7,8^ In the clinical trial, samples were collected and handled more carefully than in the routine TB programme. Challenges of sample quality and contamination are likely greater for sputum samples collected programmatically, whereby samples are kept at ambient temperature until processed in clinical laboratories, and environmental mean temperatures are 26–28°C.^9^ Our study included only samples collected at PNG’s largest TB treatment centre and handled outside of routine processes with cold storage until samples could be refrigerated in the laboratory. Samples were transported directly from Port Moresby to Melbourne. The expansion of molecular WHO-recommended rapid diagnostics (mWRDs) in PNG has enhanced the detection of multidrug-resistant/RR-TB (MDR/RR-TB) across various provinces.^10^ This increases sample numbers needing air transport to Port Moresby before shipment to Australia, prolonging storage and likely unrefrigerated transport. Contamination rates in routine practice in PNG are likely high. Reducing contamination rates and increasing yield from culture could impact treatment outcomes for people with MDR/RR-TB in PNG.

We found higher contamination in self-collected samples. These had unknown but likely unrefrigerated storage and transportation conditions to the TB clinic. Higher contamination with home collection was found in a study of sputum collection techniques.^11^ Contamination rates can be reduced through standard operating procedures (SOPs), education on sputum collection techniques, rinsing the mouth with water before sample collection and strengthening systems to preserve cold-chain, including refrigerating immediately after collection.^11,12^ Participants were coached on sample collection, but some may not have followed all the instructions during self-collection. However, there was no association between sputum purity and contaminated culture results. Contamination rates were highest in young adults (18-24 years). Older adolescents and young adults are a known high-risk group for bacteriologically positive pulmonary TB in PNG,^2,3,13^ and may require more attention and targeted education and support to optimise sputum quality.

Culture contamination was associated with delays in time from sputum collection to culture inoculation. Practices to reduce contamination commonly focus on laboratory strengthening, including standard protocols for sputum decontamination, staff training, and the implementation of quality laboratory management systems.^14^ Our findings highlight that investment in a systems approach to sputum transportation quality is essential to reducing contamination. In PNG, issues include socio-economic factors impacting patients’ ability to transport self-collected sputum, centralised collection locations, and shipments commonly delayed.

Time delays without refrigeration as short as 3 days have been reported to reduce yield and increase contamination rates.^15^ Our study showed contamination from a minimum of three days, which increased substantially with transportation delays past 7–10 days, showing the need to reduce delays to referral laboratories to a maximum of 7 days, ideally less than 3. The MDR/RR-TB referral pathway in PNG entails Xpert-confirmed RR-TB sputum from all provinces being sent to CPHL before shipping to the Queensland Mycobacterial Reference Laboratory (Brisbane, QLD, Australia).^3,4,10^ This pathway makes the 3–7 days ambition almost impossible. It is essential to strengthen sputum transport systems and culture facilities at CPHL, which was performing TB culture from January 2017^16^ until December 2021 but ceased due to laboratory infrastructure challenges.

Routine diagnosis of TB in PNG is performed with two spot sputum collections 30-60 minutes apart, which are then transported to the laboratory for Xpert MTB/RIF or Ultra testing. Xpert MTB/XDR testing was introduced in April 2023 and available in the 22 provincial hospitals by the end of 2023, enabling rapid molecular detection of mutations associated with resistance to isoniazid, fluoroquinolones, second-line injectables (amikacin, kanamycin, capreomycin), and ethionamide in a single cartridge.^17,18^ However, resistance to new and repurposed drugs, including bedaquiline, linezolid and pretomanid/delamanid, recommended for the majority of MDR/RR-TB cases,^19–22^ are not detected by current mWRDs. As shown in our study and others, Xpert MTB/RIF or Ultra results are unaffected by contamination rates.^23^ Contamination is a challenge for samples needing pDST after Xpert-diagnosed RR-TB. Culture remains necessary for treatment monitoring, highlighting the importance of evidence-based approaches to reducing culture contamination rates to improve treatment and monitoring in PNG.

WHO recently (March 2024) provided guidance on the use of targeted next-generation sequencing (tNGS) to detect resistance to many drugs in a single test, unlike the current mWRDs.^20,21^ tNGS tests provide rapid genotypic DST (molecular DST) direct from sputum samples to inform treatment decisions and faster turn-around for treatment compared to pDST, but are more complex and require specialised infrastructure,^21^ which are challenging to maintain in resource-constrained settings like PNG. However, tNGS tests do not replace culture for pDST, which is needed for testing resistance to new and repurposed anti-TB drugs.^21^ Whole-genome sequencing requires initially isolating mycobacteria in culture, as direct-from-sputum techniques are not ready for programmatic implementation in resource-constrained settings.^24^

Limitations of this study include the fact that the trial was not specifically designed to investigate factors associated with culture contamination, and therefore, important potential predictors or confounders were not recorded. For example, storage conditions and duration of unrefrigerated transportation to the TB clinic were not recorded for self-collected samples. The strengths of the study include that it utilises data collected from a trial that is compliant with Good Clinical Laboratory Practice (GCLP),^25^ and culture results were provided by a reference laboratory participating in external quality assurance, with acceptable contamination rates in routine laboratory practice.

## CONCLUSION

Sputum sample transportation delay affects mycobacterial culture yield. Although sample shipment delays contribute to culture contamination, Xpert MTB/RIF results are unaffected. Stringent primary laboratory sample handling and testing procedures will ensure no contamination is introduced after sample collection. Factors associated with contamination included unsupervised self-collection of participant sputum and delays in unrefrigerated shipment of samples. Although Xpert MTB/XDR testing is available for MDR/RR-TB, pDST is still required to detect resistance to WHO-recommended second-line drugs, including bedaquiline and delamanid. While tNGS may provide a future solution for expanded drug-resistance testing, strengthening the TB laboratory network in PNG will require investment in sputum transport systems and improving pDST availability and sample preparation steps.
